# Strategies for the Use of Poly(adenosine diphosphate ribose) Polymerase (PARP) Inhibitors in Cancer Therapy

**DOI:** 10.3390/biom2040635

**Published:** 2012-12-14

**Authors:** Cecilia E. Ström, Thomas Helleday

**Affiliations:** Science for Life Laboratory, Division of Translational Medicine and Chemical Biology, Department of Medical Biochemistry and Biophysics, Karolinska Institutet, S-171 21 Stockholm, Sweden; E-Mails: cecilia.strom@scilifelab.se (C.S.)

**Keywords:** DNA repair, poly(ADP-ribose) polymerase, synthetic lethality, cancer

## Abstract

Treatments with Poly(adenosine diphosphate ribose) polymerase (PARP) inhibitors have offered patients carrying cancers with mutated *BRCA1* or *BRCA2* genes a new and in many cases effective option for disease control. There is potentially a large patient population that may also benefit from PARP inhibitor treatment, either in monotherapy or in combination with chemotherapy. Here, we describe the multifaceted role of PARP inhibitors and discuss which treatment options could potentially be useful to gain disease control without potentiating side effects.

## 1. Introduction

**Figure 1:**
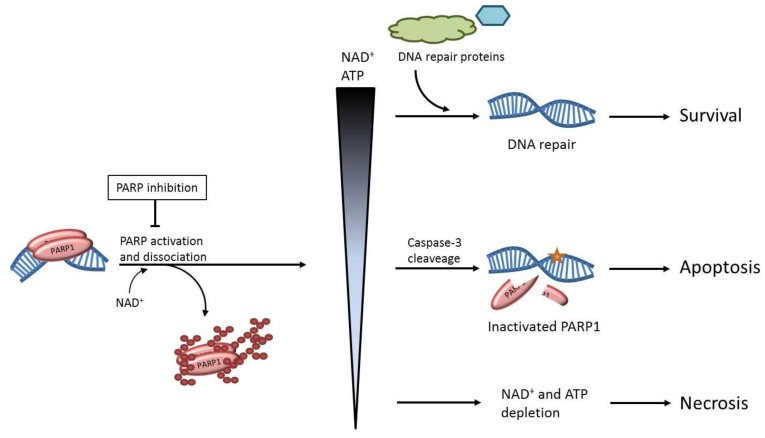
Possible outcomes of Poly(adenosine diphosphate ribose) polymerase (PARP) activation. Poly(ADP-ribose) polymerase-1 (PARP1) binds a DNA lesion and the resulting activation of the enzyme causes poly(ADP-ribose)ylation (PARylation) of PARP1 itself and other proteins. PARP1 utilizes NAD^+^ as a substrate for this modification and as the levels of activated PARP1 increase in a cell, the corresponding levels of NAD^+^ and ATP decrease. The fate of the cell after PARP1 activation depends on these intracellular levels of NAD^+^/ATP. At normal levels, cell survival is promoted, as the post-translational modification of PARP1 induces DNA repair. At lower levels of NAD^+^/ATP, PARP1 is inactivated through cleavage by caspase-3 to conserve energy for the controlled induction of apoptosis. At extremely low levels of NAD^+^/ATP, the cell rapidly dies through necrosis as a result of acute energy depletion.

Nearly 50 years ago, the first member of the large ADP-ribosyltransferase superfamily of proteins was presented [1]. This enzyme, known as poly(ADP-ribose) polymerase-1 (PARP1), can bind to DNA and it has the ability to modify itself and other proteins by the addition of ADP-ribose polymers (Figure 1). The build-up of branched ADP-ribose polymers introduces both a steric hindrance and charge repulsion of PARP1, as the large and negatively charged polymers accumulate. This modification is believed to eventually cause the dissociation of PARP1 from DNA, which enables other enzymes to reach the site of importance [2]. The addition of ADP-ribose polymers to a protein is a reversible modification that is removed within 1-2 minutes *in vivo* by the enzyme poly (ADP-ribose) glycohydrolase (PARG) [3].

The post-translational modification by PARP1 requires the respiratory co-enzyme nicotinamide adenine dinucleotide (NAD^+^) as a source of ADP-ribose and the resulting signal has been shown to affect numerous cellular processes such as DNA repair, transcriptional regulation and chromatin remodelling [4,5]. The substrate of PARP1 also provides a link between large amounts of DNA damage and cell death, as excessive activation of the enzyme leads to depletion of cellular NAD^+^, impaired ATP production and finally the induction of necrosis [6,7]. Based on this mechanism, PARP inhibitors provide potential therapies for a wide variety of diseases such as inflammatory conditions, diabetes complications, neurological diseases, as well as acute life-threatening conditions like stroke and myocardial infarction [8,9,10,11]. However, the most prominent clinical role for PARP inhibitors today lies within the field of oncology. 

The induction of DNA damage to kill cancer, using chemo- or radiotherapy is common and effective in disease control. However, such treatments are associated with toxic effects to non-transformed cells. Synthetic lethality arises as a combination of non-lethal genetic mutations or protein inactivations results in cell death, and by using this concept, selective DNA damage can be introduced to cancer cells owing to cancer specific mutations [12]. The first clinical study using the synthetic lethal concept was the use of PARP inhibitors in *BRCA1* or *BRCA2* mutated breast and ovarian cancer, which are intrinsically sensitive to PARP inhibition [13]. The inhibition of PARP alone is not sufficient to kill normal cells, but it results in an accumulation of lesions in the DNA and in repair-deficient *BRCA1* or *BRCA2* mutated cancers, these factors combined cause cell death [14,15]. Clinical evidence suggests that the use of PARP inhibitors is not restricted to *BRCA1* or *BRCA2* mutated cancers, but that it also targets non-*BRCA* mutated ovarian cancer [16] and can be useful in combination therapy. In this review we will discuss the role of PARP in DNA repair and address the clinical strategies that can be taken when using PARP inhibitors. 

## 2. The role of PARP1 in DNA Repair

Damage recognition is imperative for efficient DNA repair and PARP1 is one of the key proteins in single-strand break repair (SSBR), as it has the capacity to bind DNA nicks and ends [17]. The binding of PARP1 to a DNA single-strand break (SSB) induces a V-shaped bend in the DNA at the break and stimulates the activity of the enzyme, resulting in the assembly of ADP-ribose polymers primarily on PARP1 itself, but also on other repair proteins [18,19]. This modification results in the rapid relocation of repair proteins such as XRCC1, and eventually causes the dissociation of PARP1 from the DNA, which allows for a continuation of SSBR [2,20,21]. As opposed to its role in SSBR, the participation of PARP1 in base excision repair (BER) of small single-base damages in the DNA has been disputed by us and others, but the enzyme is known to be activated by at least a subset of SSB intermediates produced through the BER pathway [22,23]. PARP1 itself appears to be redundant for BER to be completed both *in vitro* and *in vivo*, but instead it is suggested that it captures SSB intermediates that have become uncoupled from the repair pathway. The binding of PARP1 may stimulate the repair of these intermediates, as well as constituting a cellular protection from excessive DNA damage by sequestering these potentially toxic SSB intermediates until they can be repaired [24].

**Figure 2:**
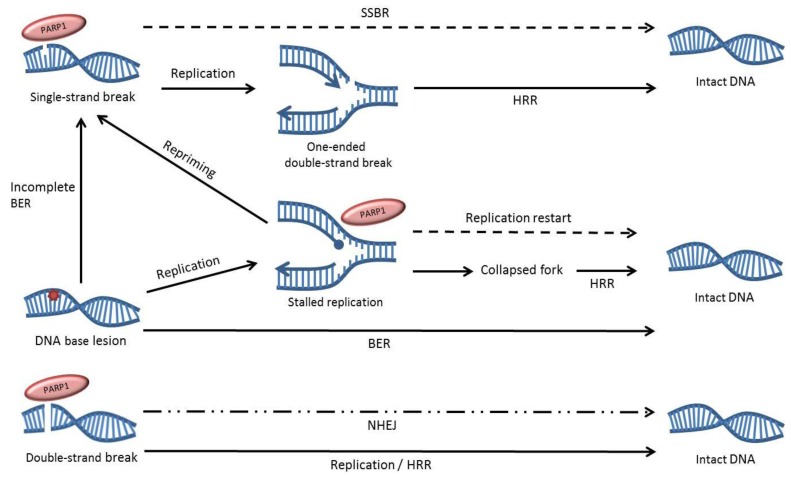
Overview of the role of PARP1 in DNA repair. PARP1 can bind to DNA single-strand breaks, double-strand breaks and replication fork structures and influences the repair of these lesions. Dotted lines indicate pathways that are impaired by PARP inhibition. The dotted and dashed line represents the partial contribution of PARP1 to non-homologous end joining (NHEJ), as it only appears to be involved in a backup form of the repair pathway.

In addition to binding SSBs, PARP1 has a strong affinity for double-strand breaks (DSBs) and has been suggested to be involved in their repair as well [17,25]. Homologous recombination repair (HRR) and non-homologous end joining (NHEJ) are the two main pathways for repair of DSBs in mammalian cells and they differ both in speed, accuracy and presence during the cell cycle. HRR requires a homologous DNA sequence for its slower, essentially error-free repair and PARP1 activity may have an indirect role in the regulation of this repair process, as unrepaired SSBs cause a build-up of recombinogenic substrates when hit by the replication machinery during the S-phase of the cell cycle. These substrates are constituted of one-ended DSBs that trigger HRR, but we and others have shown that the HRR process itself does not require PARP1 for its execution [26,27]. NHEJ is the predominant repair pathway for DSBs in mammalian cells and despite its elevated level of inaccuracy it is rapid and active throughout the cell cycle. Initially it was thought that NHEJ was driven only by DNA-dependent protein kinase (DNA-PK), but today there are accumulating reports of an alternative PARP1-dependent form of this repair pathway [28,29,30,31]. Both DNA-PK and PARP1 act as sensors of DSBs in the genome, and instigate repair by binding to the break and recruiting different downstream repair proteins. DNA-PK driven NHEJ relies on factors such as XRCC4 and DNA Ligase IV and it appears to be the preferred repair pathway in normal cells, while PARP1 instigates a backup pathway that includes the MRN complex, XRCC1 and LigIIIα [29,32,33,34,35]. Subunits of the DNA-PK protein complex interact closely with PARP1 and have been shown to suppress Figure 2 the backup NHEJ process [36,37,38,39]. 

PARP1 can also bind other types of DNA ends, such as stalled replication fork structures, and the enzyme has been shown to have an active role in the restart of stalled replication forks where it was suggested to bind and protect the fragile stalled fork structures and mediate their repair [27,40,41]. We have previously showed that PARP1 is hyperactivated when HRR is incapacitated by protein depletion, indicating a compensatory role of the enzyme [42]. Also, the activation of PARP1 occurs at replication forks, further pointing to a role of the enzyme in removing replication-associated DNA damage [40]. In addition, HRR defective cells are sensitive to the alkylating agent methyl methanesulfonate (MMS), which was shown to efficiently stall replication forks in a manner independent of SSB levels [43,44]. 

## 3. The Effects of PARP Inhibition on DNA Repair

PARP1 is not an essential protein and *PARP1*^−/−^ mice are both viable, fertile and when crossed with p53 mutant mice show delayed cancer onset [45,46,47]. The reason PARP1 is non-essential could be due to overlapping functions of PARP2, another member of the large ADP-ribosyltransferase superfamily of proteins. PARP2 is responsible for approximately 10% of the total PARP activity upon formation of SSBs and the *PARP1*^−/−^ *PARP2*^−/−^ double knockout is embryonic lethal in mice [48,49]. However, most PARP inhibitors inhibits both PARP1 and PARP2, and the side effects from this inhibition appear to be mild in both mice and humans, suggesting that the embryonic lethal effect seen in double knockout mice stems from specific problems that occur during the development, and not general survival defects [13,50]. It should be noted that removing PARP1 has been shown to cause different effects as opposed to inhibiting the enzyme [22,51]. Most PARP inhibitors target the catalytic site of the enzyme and thereby block the binding of its substrate, NAD^+^ [52]. This does not affect the binding of PARP1 to DNA ends, but prevents PAR-synthesis and leads to the enzyme being trapped on DNA. As a result, inhibition of PARP not only blocks its signalling, but the inactivated enzyme forms a steric block that may prevent access for repair proteins to the damaged site or cause problems for other processes on the DNA, such as replication. 

The precise molecular mechanism behind the killing of HRR deficient cells by PARP inhibition is not fully elucidated, but the sequestration of PARP on DNA may be important for the sensitisation of HRR defective cells [53]. An early model proposed that PARP inhibition delays SSB repair and results in the subsequent formation of recombinogenic DSBs as the replication fork runs into the unrepaired SSBs. There is a clear accumulation of SSBs in PARP inhibited cells enduring excess DNA damage induced by oxidizing or methylating agents, but it cannot be seen in untreated cells [22]. Instead, the background level of SSBs remains low after PARP knockdown or inhibition, regardless of BRCA2-status [42,54]. Also, depletion of the scaffold protein XRCC1 does not sensitise BRCA2-defective cells [55], indicating that the synthetically lethal effect seen with PARP inhibition in HRR deficient cells is separated from a blocked SSB repair of endogenous lesions. To complicate the story further, we have found a synthetic lethal effect of PARP inhibition in a XRCC1 defective hamster cell line [22]. The underlying reason for this is unknown, but finding out is most likely significant for further understanding of the molecular mechanisms causing PARP inhibitor sensitivity. It is unlikely that the observed lethality is caused by effects on SSB repair and BER alone, as there is no observed accumulation of SSBs after as long as 48 hours of PARP inhibition in these cells. More likely, this reflects the functions of these two proteins in separate pathways. 

As mentioned above, PARP1 also has functions in the backup NHEJ pathway and in the restart of stalled replication forks. It is possible that the so called backup NHEJ has a more prominent role in the repair of replication-associated lesions and that it is in part complementary in function to HRR, indicating that lesions induced by PARP inhibition are repaired by HRR during replication. Conversely, a deficiency in HRR might result in lesions that require PARP1-dependant NHEJ to be resolved. The toxic genome instability caused by PARP inhibition in BRCA2 defective cells is dependent on a functional DNA-PK dependent NHEJ, indicating a regulation of this pathway by PARP1 [55]. In addition, DNA-PK subunits have been shown to have the capacity to affect both HRR and the PARP1-dependent NHEJ [39,56,57]. 

In normal cells, the presence of both the NHEJ pathway driven by DNA-PK and the HRR pathway provides functional repair of DSBs throughout the cell cycle, even in the absence of PARP1 activity. In a study performed on breast cancer cell lines, PARP inhibition was presented as a possible novel therapeutic strategy to treat cancers that are resistant to anti-oestrogen treatment, based on the involvement of PARP1 in the alternative NHEJ pathway [58]. It was shown in both cancer cells and tumours that a lack of oestrogen and progesterone receptors correlated with decreased levels of proteins found in the DNA-PK dependent NHEJ pathway and increased levels of proteins in the backup pathway, such as PARP1. The inhibition of PARP1 was shown to sensitise these therapy-resistant cancer cell lines, and the effect was increased by the combination with a DNA ligase inhibitor. 

## 4. Potential Synthetic Lethal Interactions

Various human cancers display an overexpression of PARP1, which may be a reflection of the increased levels of DNA damage in cancer cells, often caused by oncogene-induced replication stress [59]. The deregulation of PARP1 is likely connected to its roles in different DNA repair processes as many cancer cells suffer from destabilised genome integrity. As demonstrated in BRCA1/2 deficient cells, HRR is essential to resolve the lesions presented during PARP inhibition [60]. Therefore, components of the HRR pathway are in general important for survival during PARP inhibition and several studies have confirmed this. For example, the depletion of RAD54, BLM, WRN and XRCC3 has been shown to sensitise cells to PARP inhibition, as well as *PALB2* and *RAD51D* loss of function and deficiency in Mre11, NBS1, RAD51 and RPA1 [42,61,62,63,64]. In addition, proteins that are not directly involved in HRR but rather effect the HRR status of a cell are believed to contribute to PARP inhibitor sensitivity. One example would be the genes encoding proteins that control the regulation of *BRCA1* gene expression [65]. DNA damage signalling proteins are also implicated in conferring PARP inhibitor sensitivity. A signalling cascade is initiated through the activation of the kinase ATM by DSBs in the DNA or replication stress, and this in turn induces HRR and DNA damage checkpoints. Deficiency in the signalling kinases ATM, ATR, Chk1 and Chk2 has been associated with PARP inhibitor sensitivity as well as PTEN, a tumour suppressor that is often found to be inactivated in cancer cells [64,66,67,68,69]. However, the sensitivity of PTEN defective cells may not be dependent on defects in HRR [70]. One interesting finding is that PARP inhibitors selectively sensitise CDK1 compromised cancer cells, but not CDK1 compromised normal cells [71]. One reason for the selective sensitisation of the cancer cells is likely owing to the high amount of replication stress in the cancers. The different roles of PARP1 in processes other than HRR have also been investigated in order to find synthetic lethal interactions that can be exploited for new cancer therapies. Based on the role of PARP1 at replication forks, a recent study showed a sensitisation of human cancer cells when the SMC1, SMC3 or RAD21 subunits of the cohesion complex were knocked down by siRNA in combination with PARP inhibition [72]. Mutations in these genes have been found in colon cancer tumours. 

Most of the genes mentioned above confer a sensitisation to PARP inhibition and may not be absolutely essential for survival, *i.e.*, not truly synthetically lethal. Therefore, monotherapy with PARP inhibitors will not be possible for all cancers, but combined with DNA damaging agents this provides a novel treatment strategy that can widen the therapeutic window for many forms of cancer and increase overall survival. 

## 5. Strategies for Using PARP Inhibitors in the Clinic

It is very clear that there is a benefit of using PARP inhibitors as a monotherapy in breast or ovarian cancer patients with mutations in *BRCA1* or *BRCA2* [13,16]. Interestingly, some non-*BRCA* mutant ovarian cancers also respond well to PARP inhibitors. This can potentially be explained by a general downregulation of HRR pathways in ovarian cancer, owing to epigenetic silencing of the Fanconi’s anaemia pathway [73] or to mutations in HRR genes other than *BRCA1* or *BRCA2*, e.g., *BARD1*, *BRIP1*, *CHEK2*, *MRE11A*, *MSH6*, *NBN*, *PALB2*, *RAD50*, *RAD51C*, or *RAD51D* [74,75]. Hence, further genetic testing may reveal a larger cohort of patients that can be selected for PARP inhibitor therapy. This should not be restricted to breast and ovarian cancers as also a *BRCA*-mutated prostate cancer patient has demonstrated a partial response to PARP inhibitors [13]. To avoid cancer, many *BRCA*-mutant carriers often opt for disfiguring mastectomy and/or debilitating oophorectomy at a reproductive age. As many PARP inhibitors have mild side effects, they present a complement to mastectomy and oophorectomy, as they may be used as preventive medicine to delay or prevent disease, as well as to increase the reproductive potential of affected individuals. 

**Figure 3:**
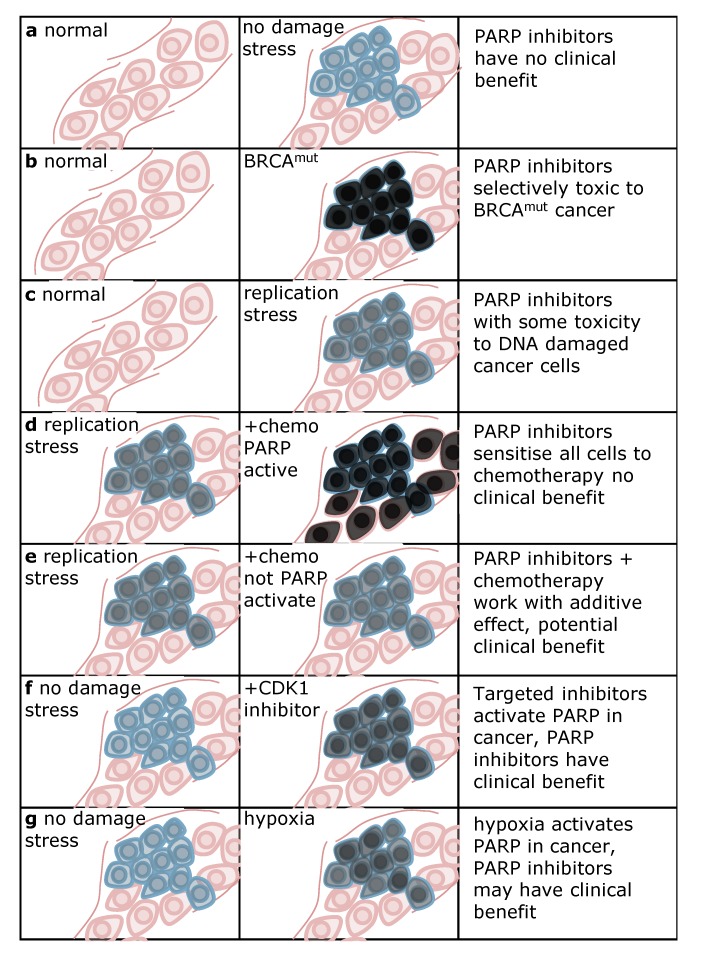
Differential PARP activity between normal and cancer tissue determines clinical benefit with PARP inhibitors. Pink indicates non-malignant tissue, blue indicates cancer tissue. Grey staining in cells denotes intensity of PARP activation, black indicating the highest activity. **(a)** In tumours without extensive load of DNA damage stress there is little activation of PARP and hence PARP inhibitors will have little clinical benefit. **(b)** PARP is highly activated in *BRCA*^mut^ cancer, providing selective toxicity to cancer cells. **(c)** Oncogene-induced replication stress may activate PARP and provide some improvement in therapeutic index with regard to toxicity to cancer cells. **(d)** PARP activating chemotherapy may close the potential therapeutic window and instead result in increased side-effects to non-malignant tissues. However, if PARP activating chemotherapy can be delivered selectively to cancer cells, improved clinical benefit may be a result. **(e)** Chemotherapy that does not activate PARP may give an additive effect and a potential clinical benefit **(f)** Targeted therapy may activate PARP in cancer cells which can make PARP inhibitors selectively toxic to cancer cells, such as the case with CDK1 inhibitors [71]. **(g)** Hypoxia impairs homologous recombination repair (HRR), rendering these cells sensitive to PARP inhibitors [83].

Currently, most PARP inhibitor trials are combined with chemotherapy in the metastatic setting. As PARP1 is important for effective DNA repair, the cytotoxic effects of chemotherapy are likely enhanced by inhibiting PARP-mediated repair. The critical issue is whether the combination of chemotherapy and PARP inhibitors will be selectively toxic to cancer cells without causing dose-limiting side effects to non-malignant cells. Thus, it is important to understand why PARP inhibitors selectively kill cancer cells in the first place. There are many functions of PARP1 and PARP inhibition traps the enzyme onto DNA, as discussed above. It is not entirely clear why PARP inhibitors kill HRR defective cells, but as PARP1 is hyperactivated and trapped on the DNA in these cells a substrate for HRR is likely formed. In tumours where there is little DNA damage, PARP1 is likely not activated and PARP inhibitors are not going to give any significant clinical benefit (Figure 3a). Hence, the selectivity to kill cancer cells is likely derived from a high amount of DNA lesions and high PARP activity in the absence of HRR (Figure 3b). HRR status and PARP activity are important aspects when discussing PARP inhibition as cancer treatment, and some strategies to improve these therapies can be derived from this. Increased PARP activity or altered HRR status may improve the clinical outcome when done selectively in cancer cells.

PARP activity can be increased in a number of ways. In fact, PARP inhibitors may even be beneficial in cancer cells with functional HRR, because of oncogene-induced replication stress that may trigger PARP activity (Figure 3c). However, the PARP activity induced after replication stress in cancer cells is likely lower than after loss of HRR and hence the toxic effects with PARP inhibitors may not be sufficient to delay tumour growth. 

In general, when an exogenous DNA damaging agent (chemotherapy) is used, there will likely be an induction of PARP activity in both normal and cancer cells (Figure 3d). In such a scenario, PARP will be needed to spare both cancer and normal cells and the selective toxicity to cancer cells is lost. Hence, the chemotherapies that trigger PARP activity may not benefit from combinations with PARP inhibitors, at least not in theory. Alternatively, if a DNA damaging agent that activates PARP could specifically induce damage in cancer cells only, it may be very useful in combination with PARP inhibitors; radiotherapy would be one such example. Importantly, PARP1 is not involved in repair of all types of DNA damage. Chemotherapy that has in itself some cancer selectivity, but not in activating PARP itself, may be combined with a PARP inhibitor and the benefit from both can be used to achieve more selective treatment of cancers (Figure 3e). Thus, the lack of sufficient toxicity with PARP inhibitor alone may be helped by combining with targeted chemotherapy to improve the therapeutic index. Recently, it was also demonstrated that CDK1 inhibitors can be used in combination with PARP inhibitors to selectively kill cancer cells and spare normal cells [71] (Figure 3f). It is not entirely clear why cancer cells are selectively targeted, but it would be unsurprising if PARP activity is selectively increased in these cancer cells. As mentioned before, various cancers are known to display an overexpression of PARP1 and some may therefore be inherently more sensitive to PARP inhibition than non-malignant cells [76,77,78,79]. 

Similarly to increased PARP activity, the HRR status has also proven important for the outcome of PARP inhibition. Agents like the PI3K inhibitors may downregulate BRCA1/2 and HRR and sensitise cells to PARP inhibitors [80,81,82]. Further combination should be tested to determine which strategies will be most useful with PARP inhibitors. Finally, hypoxia in the tumour microenvironment affects HRR and increases PARP activity which can be used in a contextual synthetic lethality approach to selectively cause DNA damage in hypoxic cells [83] (Figure 3g). The strategies exploiting the tumour microenvironment could theoretically be used in combination with anti-angiogenesis treatments to potentially improve clinical outcome. 

## 4. Conclusions

It is clear that the initial model, where PARP inhibitors impair SSB repair and cause replication-associated DSBs that are toxic in the absence of BRCA-mediated HRR, is insufficient to describe the synthetic lethality observed between PARP and BRCA proteins [53]. The role of PARP in DNA repair and at replication forks is complex. When it comes to the use of PARP inhibitors in the clinic, we suggest that the underlying PARP activity in the tumours should be monitored in order to predict the efficacy of the treatment and if a satisfactory therapeutic index is obtained. 
